# System-Size Dependence of Helix-Bundle Formation for Generic Semiflexible Polymers

**DOI:** 10.3390/polym8070245

**Published:** 2016-06-27

**Authors:** Matthew J. Williams, Michael Bachmann

**Affiliations:** 1 Soft Matter Systems Research Group, Center for Simulational Physics, The University of Georgia, Athens, GA 30602, USA; bachmann@smsyslab.org; 2 Instituto de Física, Universidade Federal de Mato Grosso, 78060-900 Cuiabá (MT), Brazil; 3 Departamento de Física, Universidade Federal de Minas Gerais, 31270-901 Belo Horizonte (MG), Brazil

**Keywords:** semiflexible polymers, structural phases, helix bundles, Monte Carlo simulation

## Abstract

Helical polymer bundles are an important fixture in biomolecular systems. The particular structural geometry of helix bundles is dependent on many factors including the length of the polymer chain. In this study, we performed Monte Carlo simulations of a coarse-grained model for helical polymers to determine the influence of polymer length on tertiary structure formation. Helical structures of semiflexible polymers are analyzed for several chain lengths under thermal conditions. Structural hyperphase diagrams, parametrized by torsion strength and temperature, are constructed and compared.

## 1. Introduction

Helical geometries are a ubiquitous element of biological polymer structures. Understanding the influences on tertiary structure formation in helical molecules is an important goal in the study of biomolecular systems. In such mesoscopic systems, structural geometry and stability are greatly influenced or even dominated by finite-size effects. The study of generic and specific conformational and thermodynamic properties of finite systems is therefore of utmost importance.

Helical structures in biological systems are generally considered to be a result of hydrogen bonding along the polymer backbone or by an ordering principle such as many-body constraints [1,2,3]. In a generic polymer simulation, helical order can be easily induced by the inclusion of torsional restraint potentials in coarse-grained models [4,5]. Helix formation can be described as a one-dimensional Ising-like transition [6,7]. Since transitions in these decidedly finite systems are not considered to be phase transitions in the thermodynamic sense [8,9], alternative approaches to understanding structural transitions have to be employed in order to gain new insight into their structure and behavior [10,11,12,13,14].

Effective potential models are useful for the study of cooperativity in complex polymer systems [15,16,17,18]. Helical polymers have been successfully explored using such models [19,20,21,22]. In addition to the properties of helical secondary-structure formation, there has also been a growing interest in understanding tertiary helix-bundle geometries and transitions [23,24,25,26,27,28,29,30,31,32,33]. Macromolecular structure and function depend on the length of the polymer and differences in system size can also lead to a qualitative change in essential features of structural organization [34,35]. Helical polymer geometry and bundling typically exhibit significant length dependence as well [36,37,38].

By analyzing an entire class of helical polymers at several lengths, we specifically investigate the influence of the system size on helical structural phases of semiflexible polymers. For this purpose, extensive replica-exchange (parallel tempering) Monte Carlo [39,40,41,42,43] simulations of a coarse-grained model for helical polymers [5,32] were performed.

## 2. Model and Results

We investigate how the stability of helix segments and the type of bundles in semiflexible helical polymers with bending restraint [5,32] depends on the system size. For this purpose, Monte Carlo computer simulations of a simplified coarse-grained polymer model were performed. In the following, we describe the model and discuss the results of our study. The simulation method is briefly summarized in the “Materials and Methods” section at the end of the paper.

### 2.1. Model for Helical Semiflexible Polymers

For the sampling of homopolymer chains with a propensity for helical structure formation, we introduce a coarse-grained model for a linear chain of monomers. Adjacent monomers are linked by elastic bonds and non-bonded interaction is governed by van der Waals forces. A bending restraint is introduced to reduce the flexibility of the chain and a torsional restraint supports the formation of helical segments.

The interaction between the bonded monomers is modeled by the finitely extensible nonlinear elastic (FENE) potential [34,44,45]. For a pair of bonded monomers, separated by a distance *r*, it is given by

(1) $$v_{\mathrm{FENE}}\left(r\right)=\log\left\{1-{\left[\left(r-r_{0}\right)/R\right]}^{2}\right\}.$$

The bond length associated with the minimum potential $r_{0}$ sets the length scale and is chosen to be unity. The maximum deviation from this value is $R\equiv \left(3/7\right)r_{0}$. Any separation of two bonded monomers beyond the stretching limit ($r>r_{0}+R$ or $r<r_{0}-R$) is specifically forbidden.

Non-bonded monomers separated by a distance *r* interact according to the Lennard-Jones (LJ) potential,

(2) $$v_{\mathrm{LJ}}\left(r\right)=\left\{\begin{array}{cc} 4\left[{\left(\sigma /r\right)}^{12}-{\left(\sigma /r\right)}^{6}\right]-v_{c}, & \mathrm{if}\;r<r_{c},\\ 0, & \mathrm{otherwise}. \end{array}\right.$$

We set $\sigma =2^{-1/6}r_{0}$, where $r_{0}$ is the length scale introduced above. The LJ potential is only nonzero for pairs of non-bonded monomers which are separated by $r<r_{c}$, improving the simulation efficiency greatly. The potential shift $v_{c}$ is necessary to avoid a discontinuity at the cut-off and therefore to satisfy $v_{\mathrm{LJ}}\left(r_{c}\right)=0$.

**Figure 1:**
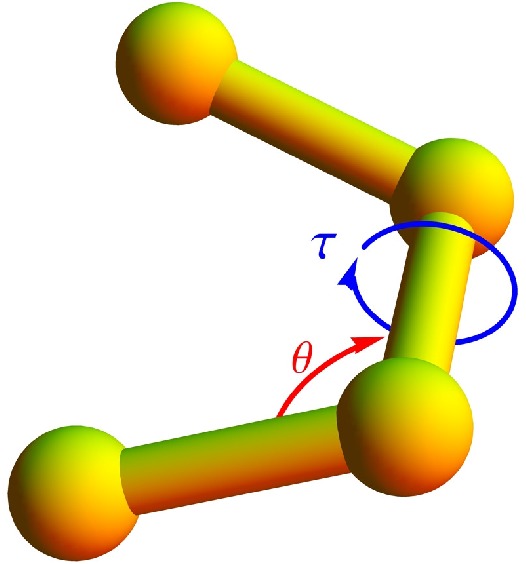
Any two adjacent bonds define a bending angle represented by *θ*. Four monomers forming three bonds are required to define the torsion angle *τ*.

A bending potential is included, based on the angle formed by two successive bonds (see Figure 1),

(3) $$v_{\mathrm{bend}}\left(\theta \right)=1-\cos\left(\theta -\theta_{0}\right);$$

where *θ* is the bending angle and the reference angle is chosen to be $\theta_{0}=1.4$. It has turned out that bending restraints are essential to stabilize helical structures [5,32] and by this, biomacromolecular matter in general.

Eventually, a potential associated with torsion angles is introduced to facilitate the formation of helical order. The torsion potential is used in the form [4]

(4) $$v_{\mathrm{tor}}\left(\tau \right)=1-\cos\left(\tau -\tau_{0}\right),$$

where *τ* represents the dihedral angle formed by the two surfaces which can be constructed using four successive monomers (see Figure 1). Any deviation in *τ* away from the reference torsion angle, $\tau_{0}=0.873$, results in an increase in the torsion potential. The value of $\tau_{0}$ is chosen such that a resultant helix will have approximately 4 monomers per turn.

The total energy associated with a particular conformation is given as a combination of all four potentials, with each having its own associated energy scale. The energy of a polymer conformation X is calculated as

(5) $$E\left(X\right)=S_{\mathrm{FENE}}\sum_{i}v_{\mathrm{FENE}}\left(r_{i\;i+1}\right)+S_{\mathrm{LJ}}\sum_{i>j+1}v_{\mathrm{LJ}}\left(r_{ij}\right)+S_{\theta }\sum_{k}v_{\mathrm{bend}}\left(\theta_{k}\right)+S_{\tau }\sum_{l}v_{\mathrm{tor}}\left(\tau_{l}\right).$$

The overall energy scale is associated with the non-bonded interaction, $S_{\mathrm{LJ}}$, and is set to unity. In these units, we chose $S_{\mathrm{FENE}}=98/5$ and $S_{\theta }=200$ [5,32]. The torsion strength $S_{\tau }$ is considered a “material” parameter in our study and varied over a range of values to explore its influence on helical structure formation. Throughout the manuscript all energy units (including the torsion strength $S_{\tau }$) are represented in multiples of the basis scale $S_{\mathrm{LJ}}$ and therefore temperatures are given in units of $S_{\mathrm{LJ}}/k_{B}$, where $k_{B}$ is the Boltzmann constant. The basis length scale is given by $r_{0}$. Both scales $S_{\mathrm{LJ}}$ and $r_{0}$ are set to unity for computational reasons in our qualitative study of generic properties and need to be adjusted appropriately if a comparison with a realistic system is attempted.

### 2.2. Classification of Helical Polymer Phases

**Figure 2:**
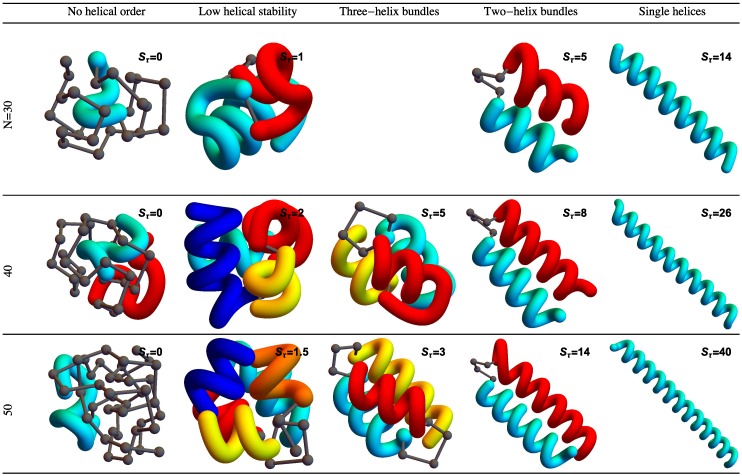
Lowest-energy structures found in our simulations for an array of system sizes and torsion strengths $S_{\tau }$. There is no stable region dominated by three-helix bundles for N=30. Colors highlight the different helical segments.

The stiffness of the helical segments exhibited by polymer structures is dependent on the strength or energy scale of the torsion potential ($S_{\tau }$). As torsion strength increases relative to attractive non-bonded interactions, helical segments stabilize into longer helices. Figure 2 shows example structures from several simulations of helical polymers for different system sizes at selected values of the torsion strength. Structures shown in this figure are the lowest-energy conformations found in our simulations and represent the putative ground states under the given conditions.

The left column shows structures generated for polymers with no torsion barrier ($S_{\tau }=0$). Without the torsion potential, helical order cannot be established and amorphous solid structures dominate the solid phase. For a nonzero, but weak torsion strength we find low-energy structures shown in the second column. While helical order and multiple helical segments are present, these segments are very flexible and unstable. Because each segment is short and flexible, they tend to couple together incoherently without aligning into predictable helix bundles. As $S_{\tau }$ continues to increase, helix segments become longer and begin to align. The third column shows three-helix bundle formation for both the 40 mers and 50 mers. For 30 mers, no stable low-temperature three-helix bundle is formed. For longer chains, individual helical segments begin to dominate over non-bonded interactions only for significantly larger values of $S_{\tau }$, which increase with system size. Effectively, in this model, three-helix bundles become more stable for larger systems as it requires a much larger torsion strength to stiffen the helical segments to the point at which they jump to the two-helix configuration. Similarly, we see the switch from two-helix to single-helix ground-state conformations, which occurs at approximately $S_{\tau }=14$ for the 30 mer, increases to $S_{\tau }=26$ for the 40 mer, and $S_{\tau }=38$ for the 50 mer.

### 2.3. Thermodynamics of Structural Transitions

**Figure 3:**
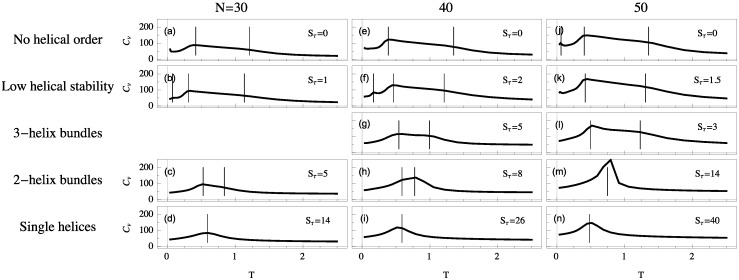
Heat-capacity curves (in units of $k_{B}$) as functions of temperature for various system sizes of semiflexible polymers with lengths N=30, 40, and 50. $S_{\tau }$ values are chosen such that lowest-energy structure types coincide in each figure row. Vertical lines mark the locations of enhanced thermal activity represented by peaks and shoulders.

For each system we simulate, the polymer exhibits a random-coil phase at high temperature. Upon cooling, these disordered structures generally collapse into a more globular liquid phase. Although structures in the liquid phase are more condensed, they still have a much higher entropy than in the solid phase, in which they reside at temperatures below the freezing transition. Despite the finiteness of the polymers we discuss here, the heat capacity is typically a good qualitative indicator for structural transitions, because extremal thermal fluctuations of the energy signal a cooperative change in the thermodynamic behavior of the system. Peaks and significant changes in monotony (“shoulders”) of these curves allow for the identification of the collapse and freezing transitions. To illustrate the different thermodynamic scenarios, heat-capacity curves as functions of temperature are shown in Figure 3 for helical semiflexible polymers of different lengths for the same $S_{\tau }$ values as used in Figure 2.

If $S_{\tau }=0$, we find that collapse (high-temperature “shoulder”) and freezing (low-temperature peak) are separate transitions. These transition signatures move towards each other as $S_{\tau }$ increases. For single-helix models, where the torsional energy dominates all other energy scales, collapse and freezing are merged into a single strong transition directly from the random-coil to the solid phase. This is the only scenario which exhibits the well-known and well-studied helix-coil transition. Collapse and freezing transitions are relatively easy to recognize, but in addition to these two consistent transitions, we find additional peaks in the specific heat below the freezing transition. These peaks correspond to solid-solid transitions which are most commonly present in disorganized helix-bundle structures and intermediate regions in which two stable helix bundle types are energetically almost degenerate. Multiple organizations are possible for short helix segments and entropically representative. In these cases, solid-solid transition signals are found representing the crossover between different helical alignments.

By comparing system sizes, we note that as polymer length increases transitions become stronger and are shifted toward slightly higher temperatures. In the following, we will analyze the qualitative transition features we identified from the heat-capacity curves more systematically by means of appropriate and specific order parameters.

### 2.4. Structural Distributions in Order Parameter Space

**Figure 4:**
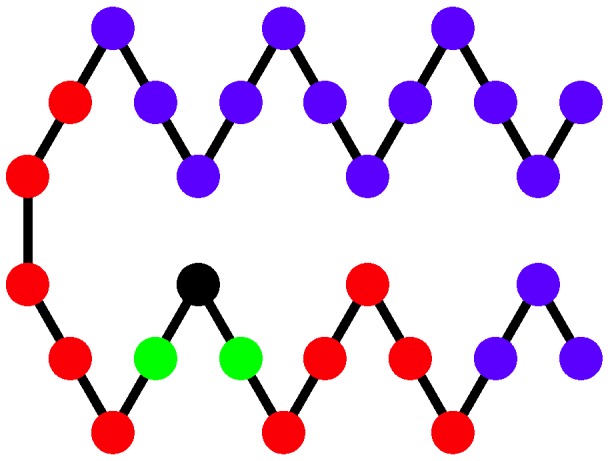
Sketch of a representative polymer structure detailing types of monomer-monomer interactions considered in this paper. The black monomer interacts via the finitely extensible nonlinear elastic (FENE) potential [34,44,45] with monomers to which it is bonded (green). Nonbonded monomers interact according to the Lennard-Jones potential. The LJ interaction between the black monomer with monomers separated by six or fewer bonds (red) contributes to $q_{1}$ while interaction with monomers separated by more than six bonds (blue) contributes to $q_{2}$.

Two structural parameters have been found to be useful in distinguishing different helical phases [5,32]. We denote by $q_{1}$ the quantity that represents the potential energy of the non-bonded interaction between monomers separated by 6 or fewer bonds and analogously introduce $q_{2}$ for the non-bonded interaction between monomers separated by more than 6 bonds as shown and described in Figure 4. Because a single helical segment exhibits no interactions between monomers which are separated by more than 6 bonds, the $q_{1}$ and $q_{2}$ values are greatly affected by the bundling of helical segments without being directly influenced by the torsion strength. This makes them an adequate set of structural parameters with resemblance of order parameters used for the discrimination of thermodynamic phases.

**Figure 5:**
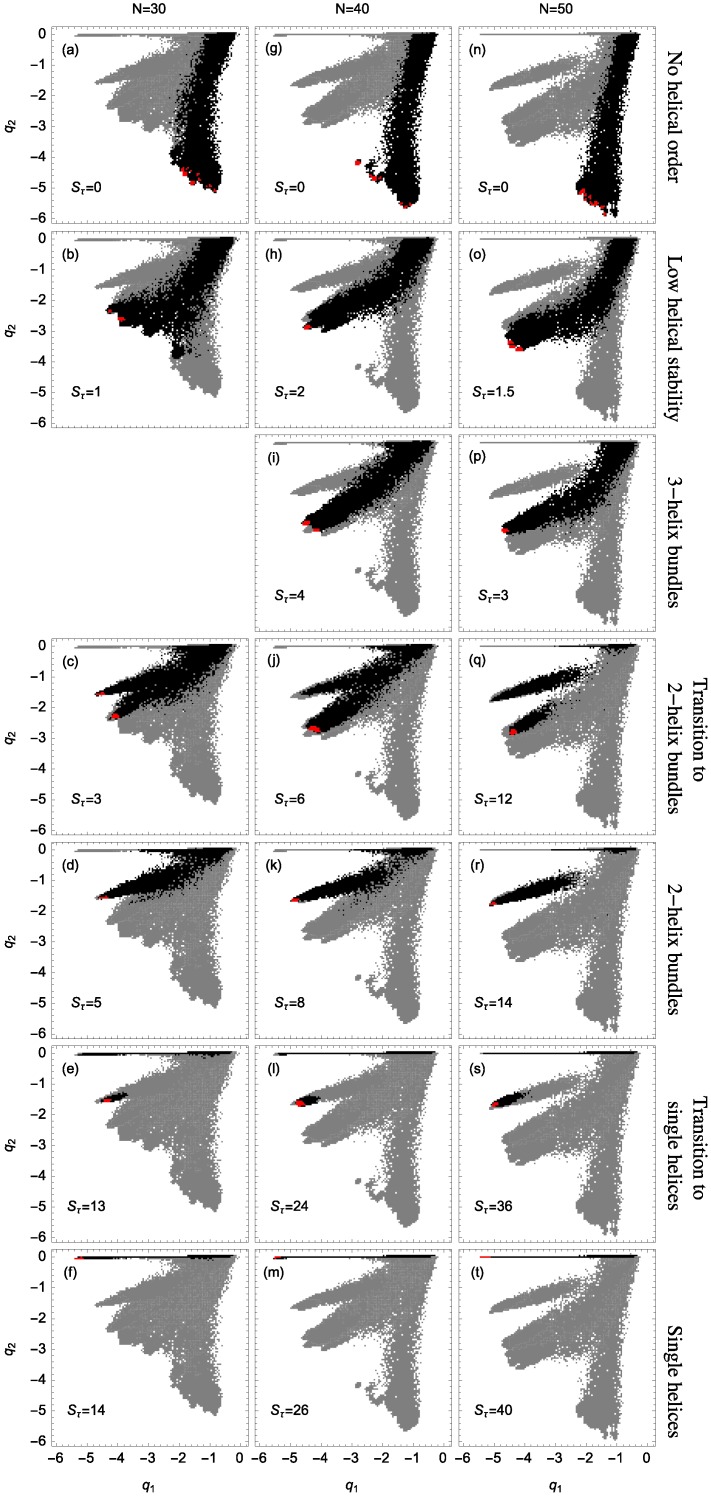
Distribution of polymer conformations in $q_{1}-q_{2}$ space for an array of values for $S_{\tau }$ and *N*. Gray regions cover all structures obtained in simulations of a single polymer length *N* across all values of $S_{\tau }$ and temperatures. Black regions highlight the part of the distribution that dominates for the specified $S_{\tau }$ value, and red regions represent only the lowest-temperature structures. Chosen $S_{\tau }$ values correspond to specific structure types. For ordered helix-bundle geometries, intermediate $S_{\tau }$ values fall into the discrete structure types shown.

The distribution of polymer conformations in $q_{1}-q_{2}$ space nicely shows separate, discrete branches associated with different structure types. Figure 5 presents the regions in which structures are present for various values of torsion strength $S_{\tau }$. In each panel, the gray areas represent the accumulated information about the structures at all simulated values of $S_{\tau }$. The black regions represent the structural distribution for an individual $S_{\tau }$ value as indicated, across all temperatures. Structures found at lowest temperatures are represented in red.

For random-coil structures, the contribution from the non-bonded Lennard-Jones interaction is almost negligible and the distribution of conformational microstates is therefore concentrated in the upper right-hand corner of the $q_{1}-q_{2}$ diagram. By cooling the system, attractive monomer-monomer interaction begins dominating over entropic effects and $q_{1}$ and $q_{2}$ values decrease, indicating that more ordered structures start forming and the distribution of states extends into one of the branches as shown in Figure 5. Depending on temperature and torsion strength, the relative contribution of $q_{1}$ versus $q_{2}$ varies in each of those branches.

Structures formed at $S_{\tau }=0$ tend to favor bonds between monomers separated by more than 6 bonds and, for low temperatures, $q_{2}$ tends to become more negative, whereas $q_{1}$ is negligible. The corresponding distribution occupies the branch associated with conformations that do not exhibit any noticeable helical order (first row in Figure 5).

As the torsion strength increases, helical segments begin to dominate the polymer structures and $q_{1}$ and $q_{2}$ behave similarly for sufficiently small values of the torsion strength (second row in Figure 5). The distributions are relatively broad which means that while the formation of helical structures become favorable, there is no dominant structure type and the conformations are rather unstable. No preferred order establishes. This instability is largest for the smallest system we studied (N=30). The reason is that out of the disordered compact states, by increasing the torsion strength slightly, symmetries develop in localized environments. This means that under these conditions rather small helical segments form and arrange in ordered bundles. Figure 5p shows that for N=50 and $S_{\tau }=3$ the dominant states already concentrate in the upper part of the branch – stable three-helix bundles form. For N=40 and $S_{\tau }=4$, we also find three-helix bundles, but the distribution is less concentrated than in the case of the 50mer and therefore represents a larger structural variety. However, ordered conformations with three helices could not clearly be identified for the 30 mer at all. The alignment tends to be quite variable and the tertiary structure is generally mixed with other structure types. The system is simply too small for a stable tertiary alignment of three helical segments. The enormous stability of the three-helix bundles for the 50 mer is evident from the fact that the dominant helical phase switches to two-helix conformations only if the torsion strength is raised to $S_{\tau }=12$. Stable two-helix conformations can already be found for the 30- and 40 mers at much lower $S_{\tau }$ values.

Qualitatively similarly for all three system sizes, there is a region of $S_{\tau }$ space which exhibits two-helix dominance. Because helix segments are longer for larger systems, the torsion strength required to stabilize two-helix bundles must increase. Note that for larger systems the transition into two-helix bundles requires the crossing of an entropically suppressed region, since for the 50 mer these structures reside on an island in $q_{1}-q_{2}$ order-parameter space. In contrast to the 30- and 40 mer, where an intermediate single-helix phase exists, the transition of the 50 mer goes directly from random coils to two-helix bundles.

For sufficiently large torsion strength, all systems exhibit a transition from random coils to single helices, representing the traditional “helix-coil” transition. Single helices are uniquely associated with most negative $q_{1}$ and almost zero $q_{2}$ values. This reflects the highly local but non-existing tertiary order of this secondary-structure type.

### 2.5. Structural Dependence on $S_{\tau }$

**Figure 6:**
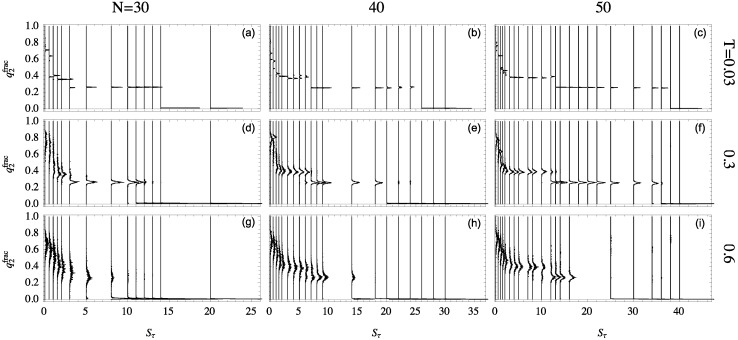
Distributions of $q_{2}^{\mathrm{frac}}$ for the semiflexible helical polymers with N=30, 40, and 50 monomers. Histograms are shown vertically with each baseline aligned with the particular $S_{\tau }$ value on the horizontal axis. Each column represents a different system size and rows represent three specific temperatures.

By projecting the phase space shown in Figure 5 onto the representative ensemble at a particular temperature, the relative quantity $q_{2}^{\mathrm{frac}}\equiv q_{2}/\left(q_{1}+q_{2}\right)$ is particularly useful for the discussion of the folding behavior of helical polymers. The distributions of this quantity are shown in Figure 6 for all system sizes studied at different temperatures. Each structure distribution corresponds to a single canonical ensemble at the given temperature. Histograms are displayed vertically with each baseline aligned with that ensemble’s $S_{\tau }$ value.

The differences in structural behavior in dependence of $S_{\tau }$ become quite apparent in this presentation, in particular, at the lowest temperature T=0.03. For the 30mer, as shown in Figure 6a, clear distinct helix bundles appear to begin at $S_{\tau }\approx 3$ where there is coexistence between globular conformations and two-helix bundles. This two-helix bundle continues to be the major type of conformation until the structural dominance crosses over to single helices at $S_{\tau }\approx 14$. For the 40 mer Figure 6b, the three-helix bundle region, from $S_{\tau }\approx 2$ to 6, is still rather unstable. The helix segment alignment and joints vary, which leads to multi-peaked, inconclusive distributions. The three-helix phase looks significantly different for the 50 mer, though, when this phase becomes essentially stable. This is also reflected by the comparatively large value of $S_{\tau }\approx 12$, where two-helix bundles become competitive.

The stability of these helix-bundle phases can also be analyzed by considering their consistency as temperature increases. The second row of Figure 6 shows structure distributions for ensembles at T=0.3, and the third row is at T=0.6. For the 30mer, the two-helix phase remains stable at T=0.3 but is nearly dissolved by T=0.6. Two-helix bundles of the 40 mer are more stable to higher temperature but still exhibit less definition than their 50 mer companions.

### 2.6. Hyperphase Diagram

Structural transitions with changes in temperature are discernible from observations of peaks and shoulders in the specific heat as a function of temperature—as seen in Figure 3. Additionally, variations in structure type upon altering the torsion strength are evident from the $q_{2}^{\mathrm{frac}}$ histograms for all $S_{\tau }$ values, as shown in Figure 6. From this data, it is possible to construct a hyperphase diagram which provides a quantitative and complete overview of the helical structural phase behavior across temperature *T* and material parameter $S_{\tau }$. For this purpose, regions of extremal thermal activity in temperature space are identified in fluctuation quantities for each individual torsion strength value. Typically, peaks of quantities like the heat capacity or the temperature derivatives of the order parameters serve as indicators for transition points. The change of the corresponding average values of $q_{1}$ and $q_{2}$ at these peak temperatures gives a first hint as to the alteration of structure types that dominate the regions below and above the transition point. Visual inspection of structures generated at temperatures in these stable conformational phases helps to confirm the characteristic structural properties of dominant macrostates in each phase.

**Figure 7:**
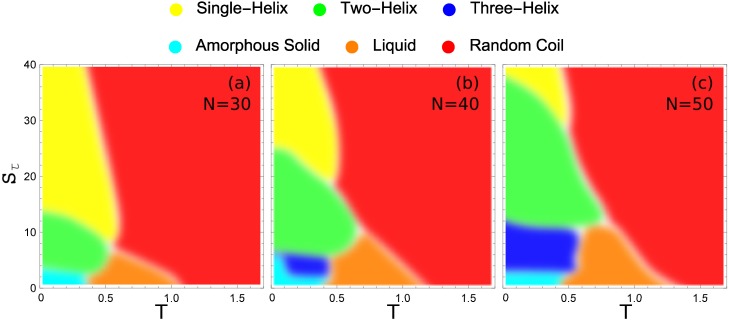
Hyper-phase diagrams for the (**a**) 30 mer, (**b**) 40 mer, and (**c**) 50 mer showing the regions of $T-S_{\tau }$ space over which each particular structure type is dominant. The color key is found above.

These hyperphase diagrams are presented in Figure 7 for the three different system sizes we compare in this study. Each major conformational phase is represented by its unique color and the color key is given above the phase diagrams. In all cases, the largest region in the given parameter space is covered by random-coil structures. There is also a liquid phase that increases slightly with system size. Also common to all system sizes is a small region at almost negligible values of the torsion strength, where the polymers fold into a compact structure with much smaller entropy than in the liquid phase. We call these structures amorphous solids, because they do not exhibit any local order and are metastable or potentially glassy. This is the regime of semiflexible polymers without torsion restraint. Apparently, no helical structure can favorably form without confining the torsion angles.

Figure 7a shows the hyperphase diagram for the 30 mer. It exhibits clearly separated phases, despite the small system size. If the torsion barrier is sufficiently strong ($S_{\tau }\geq 14$) the system exhibits only a single transition, directly from random-coil structures to single-helix configurations. This is the prominent and well-studied helix-coil transition.

As the torsion strength decreases, we reach a domain (approximately $8<S_{\tau }<13$) in which there are two distinct organized structure types with a solid-solid like transition between them. In this region, two-helix bundles are the lowest-energy structures found. Above a distinct solid-solid transition temperature, the energy benefit of the two-helix bundle gives way to the entropic suppression of these more constrained structure types. The system transitions to the predominantly single-helix configuration at this point. Below $S_{\tau }\approx 8$, there is insufficient torsion restraint to form single-helix structures. Direct transition from two-helix bundles to random-coil structures is observed between torsion strength of $S_{\tau }\approx 7$ and 8.

While there are very clear regions in which two-helix and single-helix structures are formed, when the torsion strength is very small the stiffness of helical segments is insufficient to stabilize helix bundles and non-bonded contacts among a maximum number of monomers are locally favored. For $S_{\tau }=4$, there is a solid-solid phase transition between the two-helix bundles with parallel alignment of the helical segments found at higher $S_{\tau }$ values and a new structure type in which the two helices are not aligned and a long helix wraps around a shorter helix. Decreasing the torsion strength even further the helix segments become more flexible and various different globular structures with and without helical order form, depending on the torsion strength. The amorphous solid phase is a catch-all for the different solid geometries which do not belong to a dominate helix-bundle phase.

For the longer 40 mer, there are several distinct changes from the 30 mer. The $S_{\tau }$ value at which the two-helix bundle becomes a single helix has increased significantly. This increase is because the 40 mer can form a larger number of inter-helix contacts in the two-helix case than the 30 mer, giving it a greater energetic benefit for folding in half. The transition between two-helix bundles and more globular amorphous-solid or three-helix bundle phases also occurs at a higher value of $S_{\tau }$ for similar reasons. For N=40, there is a stable three-helix bundle phase that is not present for the 30 mer. This three-helix region has only a very small domain in which it is the ground-state structure. For the largest part of the $S_{\tau }$ region over which it exists, there are also energetically more favorable structures, which include three-helix bundles with non-standard alignment and four-helix bundles with variable alignment.

For the largest system investigated in our study, the 50 mer (see Figure 7c), further expansion of helix-bundle phases into larger $S_{\tau }$ domains is found. Again the transition between two-helix bundles and single helices occurs at an increased torsion strength, as well as the transition between three-helix and two-helix bundles. Of particular note is the vastly increased parameter space over which the three-helix bundle structures dominate. Not only does the three-helix phase extend to larger values of $S_{\tau }$, but it is also stabilized down to lower values of $S_{\tau }$. The reason for the extended lower bound to the three-helix phase in $S_{\tau }$ is the decreased opportunity for misaligned helix segments in the three-helix phase.

Noteworthy in all cases, there is an intermediate low-temperature region in $S_{\tau }$ space, where the system first forms a stable single helix which upon further temperature decrease is replaced by a two-helix bundle. Due to the larger number of possible conformations for the single helix (vibrational degrees of freedom), this structure type is entropically more favorable than the two-helix bundle at higher temperatures. The two-helix structures are more rigid and structurally constrained because of the strong non-bonded energetic contacts between the helical segments. Therefore, it dominates at lowest temperatures.

## 3. Materials and Methods

The simulation details were already reported elsewhere [5,32,33] and shall be reviewed briefly here. Data used in this study was generated by means of replica-exchange Monte Carlo simulations (parallel tempering) [39,40,41,42,43]. For each individual system (*N* and $S_{\tau }$ value), parallel simulations were performed with an array 32 simulation threads, each at a unique constant temperature, distributed exponentially in the interval $T_{i}\in \left[0.03,2.5\right]$, where i=1,…,32 is the corresponding thread index. In each thread, an initial random polymer configuration was continually modified by structural updates of the monomer positions. These moves were accepted or rejected according to the Metropolis criterion [46]. Every 400 updates, a replica exchange was attempted with its neighboring thread at higher and lower temperature in accordance with the detailed-balance criterion for replica exchange [40,41,42].

Two different update types are included in these simulations. Displacement updates are the most common with rare torsion updates to improve the efficiency. For each update, one of the two types is chosen at random with the displacement update weighted to occur 3×L times more often. Displacement updates simply move a single randomly chosen monomer to a new location within a box with side length $r_{d}$ centered on the monomer’s initial location. The value of $r_{d}$ is initially iteratively determined such that the acceptance rate is approximately 0.5 in all threads. During the subsequent simulation period the respective choices of $r_{d}$ are kept constant. Because our model includes a very strong bending potential and a comparatively moderate torsion potential, much of the variation between structure types depends on torsion angles. For this reason, a global update which directly changes individual torsion angles can greatly improve the efficiency of the simulation. We do this with a torsion update in which a single bond is chosen at random. Then, all of the monomers to one side of the bond are rotated around the axis collinear with the bond by the same, random angle chosen from the interval τ∈[−π,+π].

## 4. Discussion

Extending previous studies on the importance of bending and torsion restraints [5,32], we have investigated the polymer length and torsion strength dependence of structure formation processes and conformational stability for helical semiflexible polymers. We employed a coarse-grained model containing bonded and non-bonded monomer-monomer interaction as well as bending and torsion potentials. Sampling of the conformational space was done by using the replica-exchange Monte Carlo method. In our study, we explored the entire space of torsion strengths and temperature, in which significant structural transitions are observed. Canonical statistical analysis of fluctuation quantities was performed to construct hyperphase diagrams which we compared for semiflexible polymers with 30, 40, and 50 monomers.

We find that for a polymer with 30 monomers, two-helix bundle and single-helix phases are the dominant stable folded structure types. By increasing the system size to 40 monomers, moderately stable three-helix bundles are introduced and the two-helix bundle phase is expanded over a wider range of torsion strengths. Additionally, two-helix bundles remain stable up to a slightly higher temperature. Continuing to increase the system to 50 monomers drastically increases the stability of the three-helix bundle phase in both torsion strength and temperature. The 50 mer shows further stabilization of two-helix bundles in the temperature domain and further expansion in torsion strength.

By comparing system size data, generalizations can be made about the torsion strength required to generate various structure types. We find that as the system size increases, the torsion strength required to stabilize a particular structure increases. This increase can be explained by the fact that the torsion potential penalty incurred by the formation of a joint remains constant while the energetic benefit from the increased number inter-helix contacts grows in proportion to the chain lengths. With even longer chains we expect to see these trends continue. The torsion barrier required to stabilize each structure type is supposed to increase further. For the system sizes covered in this study, stable four-helix bundles with aligned helix segments are not observed. Because the energetic benefit for non-bonded interactions in bundles with a larger number of helix segments increases with the chain length, additional structure types such as four-helix bundles are not only expected to emerge, but the optimal alignment and dense packing become additional interesting aspects of future investigations of our model.

## 5. Conclusions

Our study was based on a coarse-grained model to allow for a systematic investigation of the significant features and conditions of helix-structure formation in entire classes of semiflexible polymers with propensity for helix formation. A comparison with specific, realistic systems requires the proper adjustment of the energy and length scales left open in this generic approach.
